# Supplementary material for: Effects of Thermal and High-Pressure Processing on Quality Features and the Volatile Profiles of Cloudy Juices Obtained from Golden Delicious, Pinova, and Red Delicious Apple Cultivars
Source: Foods. 2021 Dec 8;10(12):3046. doi: 10.3390/foods10123046 (PMC8701730; doi:10.3390/foods10123046)
Supplement: Supplementary file 1 [file foods-10-03046-s001.zip › foods-1474094-supplementary.pdf]

Supplementary materials

Table S1. Characterization of the fruit's volatile fraction of three apple cultivars.

Compounds		Golden Delicious			Pinova			Red Delicious		
		ppm	±SD		ppm	±SD		ppm	±SD	
1	Hexanal	1.741	0.4		1.305	0.3		1.344	0.3	
2	Heptanal	0.046	0.0		0.000	0.0		0.000	0.0	
3	2-Hexenal	1.315	0.4		1.382	0.3		1.416	0.1	
4	Octanal	0.017	0.0	a	0.000	0.0	b	0.015	0.0	b
5	2-Heptenal	0.000	0.0	b	0.056	0.0	a	0.000	0.0	b
6	Nonanal	0.059	0.0		0.043	0.0		0.049	0.0	
7	2-Octenal	0.000	0.0	b	0.038	0.0	a	0.000	0.0	b
8	Decanal	0.029	0.0		0.035	0.0		0.032	0.0	
9	Benzaldehyde	0.000	0.0	b	0.036	0.0	a	0.037	0.0	a
10	2-Nonenal	0.000	0.0	b	0.019	0.0	a	0.022	0.0	a
11	Benzenacetaldehyde	0.000	0.0		0.052	0.0		0.052	0.0	
12	Isobutyl acetate	0.000	0.0	b	0.028	0.0	a	0.036	0.0	a
13	Isoamyl acetate	0.052	0.0	b	0.710	0.1	a	0.529	0.0	a
14	(E)-2-Methyl-2-butenyl acetate	0.000	0.0	b	0.046	0.0	a	0.040	0.0	a
15	Amylbutyrate	0.000	0.0	b	0.013	0.0	a	0.017	0.0	a
16	Hexyl acetate	0.035	0.0	b	0.149	0.0	a	0.165	0.0	a

17	2-Hexenyl acetate	0.000	0.0		0.000	0.0		0.027	0.0	
18	Butylcaproate	0.118	0.0		0.025	0.0		0.026	0.0	
19	Hexylbutyrate	0.124	0.1		0.077	0.0		0.077	0.0	
20	Hexyl n-valerate	1.189	0.5		0.129	0.0		0.135	0.0	
21	Isoamylcaproate	0.050	0.0		0.060	0.0		0.063	0.0	
22	2-hexenyl butyrate	0.000	0.0	b	0.033	0.0	a	0.038	0.0	a
23	cis-3-Hexenyl 2-methylbutyrate	0.000	0.0		0.000	0.0		0.037	0.0	
24	Pentylhexanoate	0.039	0.0		0.000	0.0		0.000	0.0	
25	Isobutyloctanoate	0.000	0.0		0.013	0.0		0.012	0.0	
26	Prenylcaproate	0.000	0.0		0.000	0.0		0.018	0.0	
27	Hexylcaproate	0.792	0.2		0.000	0.0		0.137	0.0	
28	butylcaprylate	0.092	0.0		0.016	0.0		0.019	0.0	
29	2-methylbutyl octanoate	0.048	0.0		0.071	0.0		0.074	0.0	
30	(E)-2-Hexenyl hexanoate	0.000	0.0		0.021	0.0		0.019	0.0	
31	Phenylmethyl acetate	0.000	0.0		0.000	0.0		0.011	0.0	
32	Hexylcaprylate	0.023	0.0		0.000	0.0		0.000	0.0	
33	Butanol	0.030	0.0		0.000	0.0		0.000	0.0	
34	Isolamylalcohol	0.126	0.0		0.215	0.1		0.225	0.0	
35	Hexanol	0.944	0.3		0.719	0.3		0.733	0.2	
36	3-Hexen-1-ol	0.000	0.0	b	0.020	0.0	a	0.021	0.0	a
37	2-Hexen-1-ol	0.084	0.0		0.174	0.1		0.177	0.1	
38	1 Octen-3-ol	0.000	0.0	b	0.017	0.0	a	0.018	0.0	a
39	6-Methyl-5-hepten-2-ol	0.000	0.0		0.054	0.0		0.055	0.0	

40	2-Ethyl-1-hexanol	0.054	0.0		0.052	0.0		0.055	0.0	
41	PhenylethylAlcohol	0.000	0.0		0.000	0.0		0.029	0.0	
42	β-Myrcene	0.000	0.0		0.092	0.0		0.000	0.0	
43	Estragole	0.034	0.0		0.049	0.0		0.053	0.0	
44	(Z. E)-α-farnesene	0.000	0.0		0.000	0.0		0.018	0.0	
45	(E. E)-α-farnesene	4.670	1.8		1.897	0.3		2.006	0.2	
46	Sulcatone	0.184	0.1		0.141	0.0		0.146	0.0	
47	butyrolactone	0.115	0.0		0.000	0.0		0.000	0.0	
48	Dodecane	0.000	0.0	b	0.144	0.0	a	0.150	0.0	a
49	Tridecane	0.104	0.0	b	0.287	0.1	a	0.278	0.0	a

Values in same row, followed by different letters, are statistically different. One way ANOVA, Tukey's test ($p \leq 0.05$)

Table S2. Influence of cultivar and pasteurization treatments on the apple juice volatile fraction.

	Compounds	Golden Delicious						Pinova						Red Delicious						Factors		
		NT		TT		HPP		NT		TT		HPP		NT		TT		HPP		CV	TT	CV*TT
		ppm	±SD	ppm	±SD	ppm	±SD	ppm	±SD	ppm	±SD	ppm	±SD	ppm	±SD	ppm	±SD	ppm	±SD			
1	Hexanal	0,610	0,0	0,533	0,4	3,374	0,0	2,902	1,1	1,222	0,2	1,565	0,2	3,106	1,3	1,355	0,3	0,826	0,1	0,542	0,020	0,0022
2	Heptanal	0,000	0.0	0,019	0,0	0,032	0,0	0,000	0.0	0,000	0.0	0,000	0.0	0,000	0.0	0,000	0.0	0,000	0.0	0,000	0,000	0,000
3	2-Hexenal	1,099	0,0	0,908	0,3	2,101	0,1	2,567	0,0	1,983	0,1	2,383	0,2	2,785	0,1	3,120	1,0	2,264	0,6	0,001	0,606	0,047
4	Octanal	0,000	0.0	0,000	0.0	0,015	0,0	0,000	0.0	0,015	0,0	0,013	0,0	0,000	0.0	0,000	0.0	0,000	0.0	0,000	0,000	0,000
5	2-Heptenal	0,000	0.0	0,023	0,0	0,069	0,0	0,000	0.0	0,000	0.0	0,000	0.0	0,000	0.0	0,000	0.0	0,000	0.0	0,000	0,001	0,000
6	Nonanal	0,029	0,0	0,000	0.0	0,041	0,0	0,022	0,0	0,018	0,0	0,000	0.0	0,000	0.0	0,000	0.0	0,000	0.0	0,000	0,000	0,000
7	2-Octenal	0,000	0.0	0,000	0.0	0,070	0,0	0,000	0.0	0,030	0,0	0,000	0.0	0,000	0.0	0,000	0.0	0,000	0.0	0,000	0,000	0,000
8	Furfural	0,000	0.0	0,000	0.0	0,056	0,0	0,000	0.0	0,054	0,0	0,037	0,0	0,000	0.0	0,058	0,0	0,000	0.0	0,133	0,000	0,000
9	Decanal	0,009	0,0	0,010	0,0	0,031	0,0	0,020	0,0	0,030	0,0	0,036	0,0	0,023	0,0	0,033	0,0	0,014	0,0	0,167	0,274	0,149
10	Benzaldehyde	0,067	0,0	0,212	0,2	0,768	0,6	0,039	0,0	0,027	0,0	0,042	0,0	0,048	0,0	0,031	0,0	0,047	0,0	0,041	0,157	0,152
11	2-Nonenal	0,004	0,0	0,000	0.0	0,025	0,0	0,009	0,0	0,007	0,0	0,000	0.0	0,010	0,0	0,010	0,0	0,000	0.0	0,019	0,134	0,000
12	Benzenacetaldehyde	0,000	0.0	0,000	0.0	0,000	0.0	0,018	0,0	0,000	0.0	0,057	0,0	0,017	0,0	0,015	0,0	0,026	0,0	0,000	0,000	0,000
13	2,4-Decadienal	0,000	0.0	0,000	0.0	0,016	0,0	0,000	0.0	0,000	0.0	0,000	0.0	0,000	0.0	0,000	0.0	0,000	0.0	0,004	0,004	0,002
14	2,5-Dimethyl-benzaldehyde	0,000	0.0	0,000	0.0	0,000	0.0	0,014	0,0	0,000	0.0	0,000	0.0	0,018	0,0	0,000	0.0	0,003	0,0	0,000	0,000	0,000
15	Isobutyl acetate	0,011	0,0	0,025	0,0	0,035	0,0	0,052	0,0	0,102	0,0	0,068	0,0	0,048	0,0	0,091	0,0	0,011	0,0	0,000	0,001	0,005
16	Butyl acetate	0,044	0,0	0,313	0,1	0,165	0,1	0,154	0,0	0,281	0,0	0,091	0,0	0,169	0,0	0,298	0,0	0,061	0,0	0,996	0,000	0,013
17	Isoamyl acetate	0,083	0,0	0,252	0,2	0,295	0,1	0,899	0,5	1,784	0,9	0,828	0,1	0,987	0,5	1,802	0,8	0,618	0,3	0,012	0,067	0,489
18	Amyl acetate	0,000	0.0	0,000	0.0	0,033	0,0	0,057	0,0	0,069	0,0	0,031	0,0	0,060	0,0	0,076	0,0	0,000	0.0	0,020	0,150	0,045
19	Prenyl acetate	0,000	0.0	0,016	0,0	0,022	0,0	0,000	0.0	0,000	0.0	0,086	0,0	0,000	0.0	0,000	0.0	0,000	0.0	0,002	0,000	0,001
20	(E)-2-Methyl-2-butenyl acetate	0,000	0.0	0,000	0.0	0,000	0.0	0,086	0,0	0,112	0,0	0,000	0.0	0,081	0,0	0,133	0,0	0,118	0,0	0,000	0,000	0,000
21	Hexyl acetate	0,031	0,0	0,309	0,3	0,119	0,0	0,480	0,0	1,163	0,3	0,226	0,1	0,467	0,0	1,456	0,0	0,052	0,0	0,000	0,000	0,001

22	3-Hexenyl acetate	0,000	0.0	0,000	0.0	0,000	0.0	0,016	0,0	0,027	0,0	0,000	0.0	0,000	0.0	0,029	0,0	0,000	0.0	0,000	0,000	0,000
23	Butylcaproate	0,015	0,0	0,050	0,0	0,043	0,0	0,020	0,0	0,026	0,0	0,000	0.0	0,024	0,0	0,025	0,0	0,028	0,0	0,147	0,057	0,002
24	Hexylbutyrate	0,000	0.0	0,000	0.0	0,066	0,0	0,019	0,0	0,035	0,0	0,000	0.0	0,024	0,0	0,098	0,1	0,000	0.0	0,000	0,000	0,001
25	Hexyl n-valerate	0,031	0,0	0,149	0,1	0,279	0,1	0,128	0,0	0,216	0,0	0,000	0.0	0,134	0,0	0,141	0,1	0,106	0,0	0,527	0,157	0,007
26	Isoamylcaproate	0,000	0.0	0,000	0.0	0,000	0.0	0,000	0.0	0,000	0.0	0,000	0.0	0,000	0.0	0,000	0.0	0,014	0,0	0,009	0,009	0,005
27	2-hexenyl butyrate	0,000	0.0	0,000	0.0	0,000	0.0	0,000	0.0	0,000	0.0	0,000	0.0	0,000	0.0	0,000	0.0	0,008	0,0	0,006	0,006	0,003
28	Pentylhexanoate	0,021	0,0	0,059	0,0	0,171	0,1	0,032	0,0	0,031	0,0	0,023	0,0	0,035	0,0	0,054	0,0	0,061	0,0	0,033	0,031	0,034
29	butylcaprylate	0,000	0.0	0,000	0.0	0,000	0.0	0,007	0,0	0,000	0.0	0,000	0.0	0,012	0,0	0,014	0,0	0,000	0.0	0,000	0,003	0,005
30	Ethyldecanoate	0,010	0,0	0,000	0.0	0,013	0,0	0,000	0.0	0,000	0.0	0,000	0.0	0,000	0.0	0,000	0.0	0,000	0.0	0,000	0,000	0,000
31	Phenylmethyl acetate	0,000	0.0	0,000	0.0	0,000	0.0	0,000	0.0	0,000	0.0	0,326	0,1	0,000	0.0	0,000	0.0	0,000	0.0	0,000	0,000	0,000
32	Hexylcaprylate	0,000	0.0	0,000	0.0	0,000	0.0	0,010	0,0	0,000	0.0	0,000	0.0	0,031	0,0	0,000	0.0	0,009	0,0	0,000	0,000	0,000
33	Butanol	0,020	0,0	0,071	0,0	0,055	0,0	0,050	0,0	0,000	0.0	0,000	0.0	0,063	0,0	0,000	0.0	0,000	0.0	0,002	0,007	0,000
34	Isolamylalcohol	0,078	0,0	0,132	0,0	0,162	0,0	0,303	0,0	0,000	0.0	0,308	0,0	0,319	0,0	0,000	0.0	0,132	0,2	0,090	0,001	0,003
35	Prenol/2-Heptanol	0,000	0.0	0,000	0.0	0,012	0,0	0,021	0,0	0,035	0,0	0,000	0.0	0,021	0,0	0,040	0,0	0,041	0,0	0,001	0,137	0,021
36	Hexanol	1,016	0,1	1,110	0,0	1,090	0,0	0,828	0,0	0,680	0,0	0,393	0,1	0,860	0,1	0,720	0,1	0,175	0,1	0,000	0,000	0,000
37	3-Hexen-1-ol	0,006	0,0	0,134	0,1	0,000	0.0	0,000	0.0	0,000	0.0	0,000	0.0	0,009	0,0	0,000	0.0	0,041	0,1	0,103	0,155	0,032
38	2-Hexen-1-ol	0,222	0,0	0,000	0.0	0,032	0,0	0,065	0,0	0,000	0.0	0,056	0,0	0,062	0,0	0,000	0.0	0,000	0.0	0,000	0,000	0,000
39	1 Octen-3-ol	0,005	0,0	0,025	0,0	0,033	0,0	0,000	0.0	0,020	0,0	0,015	0,0	0,007	0,0	0,016	0,0	0,000	0.0	0,015	0,004	0,033
40	1-heptanol	0,010	0,0	0,000	0.0	0,025	0,0	0,000	0.0	0,021	0,0	0,000	0.0	0,000	0.0	0,000	0.0	0,000	0.0	0,000	0,014	0,000
41	6-Methyl-5-hepten-2-ol	0,006	0,0	0,090	0,1	0,000	0.0	0,024	0,0	0,000	0.0	0,067	0,0	0,024	0,0	0,065	0,0	0,032	0,0	0,786	0,162	0,033
42	2-Ethyl-1-hexanol	0,005	0,0	0,012	0,0	0,000	0.0	0,011	0,0	0,014	0,0	0,014	0,0	0,012	0,0	0,014	0,0	0,012	0,0	0,000	0,001	0,001
43	Octanol	0,012	0,0	0,025	0,0	0,039	0,0	0,038	0,0	0,042	0,0	0,024	0,0	0,040	0,0	0,026	0,0	0,015	0,0	0,177	0,584	0,015
44	2-Octen-1-ol	0,000	0.0	0,000	0.0	0,021	0,0	0,000	0.0	0,000	0.0	0,000	0.0	0,000	0.0	0,000	0.0	0,000	0.0	0,000	0,000	0,000
45	Nonanol	0,010	0,0	0,011	0,0	0,000	0.0	0,000	0.0	0,000	0.0	0,000	0.0	0,000	0.0	0,000	0.0	0,000	0.0	0,000	0,010	0,005
46	PhenylethylAlcohol	0,000	0.0	0,000	0.0	0,021	0,0	0,000	0.0	0,000	0.0	0,000	0.0	0,000	0.0	0,000	0.0	0,000	0.0	0,000	0,000	0,000
47	Linalool	0,003	0,0	0,000	0.0	0,011	0,0	0,000	0.0	0,000	0.0	0,000	0.0	0,000	0.0	0,000	0.0	0,006	0,0	0,000	0,000	0,000

48	Caryophyllene	0,000	0.0	0,000	0.0	0,038	0,0	0,000	0.0	0,000	0.0	0,000	0.0	0,000	0.0	0,000	0.0	0,000	0.0	0,000	0,000	0,000
49	Estragole	0,010	0,0	0,024	0,0	0,037	0,0	0,051	0,0	0,074	0,0	0,068	0,0	0,053	0,0	0,077	0,0	0,010	0,0	0,000	0,003	0,001
50	(E, E)-α-farnesene	0,217	0,0	0,354	0,0	1,153	0,6	0,439	0,0	0,520	0,1	0,023	0,0	0,469	0,0	0,556	0,1	0,421	0,2	0,164	0,438	0,006
51	Sulcatone	0,041	0,0	0,087	0,1	0,135	0,0	0,050	0,0	0,076	0,0	0,077	0,0	0,048	0,0	0,080	0,0	0,035	0,0	0,074	0,029	0,047

Two way ANOVA, Tukey’s test ($p \leq 0.05$)
